# PCL retained is safe in medial pivot TKA—a prospective randomized trial

**DOI:** 10.1007/s00167-023-07634-2

**Published:** 2023-11-14

**Authors:** Nicolaas C. Budhiparama, Imelda Lumban-Gaol, Kiki Novito, Hendy Hidayat, Federico De Meo, Giorgio Cacciola, Pietro Cavaliere

**Affiliations:** 1 Department of Orthopaedic and Traumatology, Faculty of Medicine, Universitas Airlangga, Surabaya, Indonesia; 2 Department of Orthopaedics, Leiden University Medical Centre, Leiden, The Netherlands; 3 Nicolaas Institute of Constructive Orthopaedic Research and Education Foundation for Arthroplasty and Sports Medicine at Medistra Hospital, Jakarta, Indonesia; 4 GIOMI Istituto Ortopedico del Mezzogiorno d’Italia Franco Scalabrino, Ganzirri, Via Consolare Pompea, 98165 Messina, ME Italy

**Keywords:** Medial pivot prosthesis, Medial pivot TKA, PCL, PCL retention, PCL resection, PCL sacrifice

## Abstract

**Purpose:**

Medial pivot (MP) designs resemble native knee kinematics and restore the “natural” kinematics of a knee after total knee arthroplasty (TKA). However, whether to preserve or resect the posterior cruciate ligament (PCL) is still under debate. We inquired whether sacrificing the PCL would improve range of motion, functional outcomes, and limb alignment compared to preserving the PCL in TKA using medial pivot implants (MP-TKA).

**Methods:**

This prospective, double-blinded, randomized controlled trial consisted of 33 patients (66 knees) undergoing bilateral simultaneous MP-TKA. In one knee, a PCL preservation technique was performed, and in the contralateral knee, the PCL was resected. The primary outcome was postoperative range of motion (ROM). The secondary outcomes were visual analogue scale (VAS) score for knee pain at walking, Knee Injury and Osteoarthritis Outcome Score for symptoms (KOOS-S) and quality of life (KOOS-QoL), Oxford knee score (OKS), and Forgotten Joint Score (FJS), and measurement of the mechanical femoral-tibial axis (mFTA) on X-ray images. All patients were followed up for a minimum of 2 years after surgery.

**Results:**

Patients who underwent MP-TKA with PCL preservation had a similar ROM at 2 years (125.45 ± 7.00 vs. 126.21 ± 6.73, *p* = 0.65) as those who underwent MP-TKAs with PCL resection. There was also no difference in VAS score (1.94 ± 0.79 vs. 2.00 ± 0.71, respectively, *p* = 0.51), OKS (39.97 ± 2.01 vs. 39.67 ± 2.03, respectively, *p* = 0.52), KOOS-S (84.41 ± 3.77 vs. 84.19 ± 3.57, respectively, *p* = 0.92), KOOS-QoL (82.94 ± 4.76 vs. 82.75 ± 4.70, respectively, *p* = 0.84), or FJS (72.66 ± 8.99 vs. 72.35 ± 8.64, respectively, *p* = 0.76) at the 2-year follow-up. No difference in the measurement of the mFTA was found between the two groups (180.27 ± 2.25 vs. 181.30 ± 2.13, respectively, *p* = 0.59).

**Conclusion:**

This study demonstrated that both medial pivot TKA with PCL preservation and PCL resection achieved excellent results. There was no difference at the 2-year follow-up in terms of postoperative ROM, patient-reported outcomes, or radiographic evaluation.

**Level of Evidence:**

Therapeutic study, Level I.

## Introduction

Total knee arthroplasty (TKA) is a common orthopaedic procedure that aims to create a “natural knee” that has similar function and kinematics as those of the native knee. Since its inception, TKA techniques and implant designs have evolved to achieve that goal. There is a 25% unsatisfaction rate with current implant designs, cruciate-retaining (CR), or posterior-stabilized (PS) TKAs [1, 8]. CR-TKA produces abnormal translation and rotation between the tibia and femur [7, 31, 38]. PS-TKA also results in posterior femoral translation [36, 37, 40].

The concept of the medial pivot implant is to create a centre of rotation on the medial side so that the lateral side can translate anteriorly or posteriorly during extension and flexion [4, 12, 14, 25, 27]. This understanding of normal knee kinematics is based on the new concept of medial pivot implants, which was proposed in 2002. This implant has spherical medial and lateral condyles with a liner as a socket [3]. It was believed that the design of this prosthesis was a posterior cruciate-substituting prosthesis. Therefore, PCL resection is commonly performed in MP implantation [18]. Several studies have shown clinical, radiographical, and kinematical improvement with an MP implant [19, 21]. However, controversy arises regarding whether to preserve or sacrifice the posterior cruciate ligament (PCL) in medial pivot TKA [2, 11, 39]. Whether to preserve or sacrifice the PCL in medial pivot TKA is still based on surgeon preference [15, 26]. However, there is an opinion that retaining the PCL can become an obstacle in femoral rollback. Therefore, in this study, we performed this simultaneously in TKA to compare the effects of PCL retention and PCL resection on femoral rollback by evaluating patient-reported outcome measurements (PROMs), where we eliminated confounders such as differences in pain and subjective perceptions between individuals.

The primary objective of this study was to determine whether there was any difference in postoperative range of motion between patients whose PCL was resected and patients whose PCL was preserved in simultaneous bilateral TKA with medial pivot implants. It is hypothesized that sacrificing the PCL in TKA with medial pivot implants results in a better range of motion than retaining the PCL. The secondary objectives are to compare the functional outcomes and limb alignment of those 2 groups of patients treated with simultaneous bilateral TKA at the short-term follow-up. The second hypothesis was that sacrificing the PCL in TKA with medial pivot implants results in better functional outcomes and better limb alignment.

## Materials and methods

### Study design

We conducted a prospective, single-centre, double-blinded, randomized controlled trial comparing outcomes of medial pivot TKA with PCL retention and PCL resection at Medistra Hospital, Jakarta, under the approval of its institutional review board. This study complied with the CONSORT 2010 statement. Each patient signed a consent form prior to enrolment in this study. Patient recruitment began in January 2018 and continued until April 2020. All patients were followed up for a minimum of 2 years after surgery (mean 33 ± 7.1 months with a range of 24–50 months).

### Eligibility criteria

The inclusion criteria for this study were as follows: patients with symptomatic primary, bilateral varus knee osteoarthritis (minimum Kellgren–Lawrence grade 3) who required primary bilateral TKA; patients fit for surgical intervention; patients aged between 18 and 90 years at the time of surgery; patients who were able to give informed consent and agree to comply with the postoperative review programme; and patients who were able to attend follow-up visits at the clinic. The exclusion criteria for this study included the following: patients with rheumatoid arthritis, infected or septic arthritis, and other inflammatory arthritis; secondary or traumatic osteoarthritis; preexisting or congenital bony deformities; severe knee deformities (a varus or valgus deformity) greater than 15°; flexion contracture greater than 10°; patellar dislocation; a requirement for arthroplasty for fracture or previous osteotomy; underlying neurological dysfunction compromising mobility; and an inability to tolerate general anaesthesia.

### Allocation

To decide which knee the PCL was going to be preserved and which knee the PCL was going to be sacrificed, the main surgeon randomly drew a sealed envelope from a box before the surgery. The envelope contained the name of the side on which the PCL was going to be resected. Only the main surgeon and assistant knew the name of the side on which the PCL would be preserved.

### Intervention

A single experienced surgeon performed all surgeries. The implanted prosthesis was the K-MOD medial pivot implant system (Gruppo Bioimpianti, Italy). A tourniquet was used during the procedure. The operation was always performed first on the right side, regardless of the severity of arthritis, through a standard medial parapatellar approach. A tibial cut was made perpendicular to the mechanical axis using an extramedullary alignment guide. The tibial posterior slope was adjusted based on preoperative X-ray (ranged 3–7°), as anatomically precise as possible, to achieve the appropriate tension of the PCL and balancing of the flexion gap. A distal femoral cut was made in 5° of valgus using an intramedullary femoral alignment guide, and the extension gap was measured using spacer blocks. Femoral rotation and the posterior femoral condyle cut were determined using a hybrid method, a combination of the measured resection technique and gap-balancing technique, and the flexion gap was measured using spacer blocks. The posterior femoral osteophytes were excised. Subsequently, trial implants were inserted to assess the joint space; varus and valgus stability, patellar tracking, and ROM were measured; and the pull-out lift-off test[30] was performed. Patella osteophytes were excised, and none of the patella was resurfaced in either knee. Circumferential electrocautery was not performed on the patellar rim, as proven in a previous study [5]. PCL release was performed based on the result from the envelope drawn before surgery. If the knee was stable with no tightness during flexion or extension and no presence of patellar maltracking, then the final tibial and femoral components were implanted. No patellar maltracking or ligament imbalance was found in this study during intraoperative evaluation. The wound was closed, and vacuum drainage was used. The same technique was used on the contralateral knee. The patients were not aware of which knee the PCL was released from.

### Postoperative care and rehabilitation

Physiotherapy was performed as soon as the patient returned to the ward, the same day of surgery. The vacuum drain was removed at a maximum of 24 h after surgery. ROM and straight-leg raising exercises were performed continuously during the first day in the ward. A continuous passive motion device was also used. Isometric quadriceps strengthening exercises and assisted weightbearing ambulation using a walker were performed on the second day. Antithrombotic prophylaxis using an oral direct-factor Xa inhibitor (rivaroxaban) was administered to all patients for 14 days postoperatively. The postoperative care and rehabilitation were identical for both knees.

### Outcomes

We evaluated the range of motion (ROM) as the primary outcome. For secondary outcomes, we evaluated the visual analogue scale (VAS) score for knee pain at walking, Knee Injury and Osteoarthritis Outcome Score for symptoms (KOOS-S) and quality of life (KOOS-QoL), Oxford knee score (OKS), and Forgotten Joint Score (FJS). We also evaluated the mechanical femoral-tibial axis (mFTA) for radiographic evaluation as a secondary outcome. Patients were surveyed preoperatively and at the latest follow-up (minimum 2 years) postoperatively by a single orthopaedic surgeon who was not a part of the main surgical team and was not aware of the patient’s group allocations or radiograph evaluation results until the end of the study.

### Radiographic assessment

Bilateral knee and full-length lower limb standing X-rays were taken before the surgery, and bilateral knee radiographs were taken 24 h after surgery. Postoperative full-length lower limb standing X-rays were taken at the first follow-up. The mechanical femorotibial angle of both knees was measured using the full-length lower limb standing radiograph via digital image viewer software (General Electric Centricity Digital Imaging and Communications in Medicine Viewer 3.1.4., Chicago, Illinois, United States). One independent radiologist performed all radiographic evaluations twice to reduce intraobserver bias. The ICC was 0.76.

### Sample size

We used the FJS to measure the sample size, as we know that the FJS is used to assess how natural the prosthesis feels after TKA. Using a 2-tailed, 2-sample t test, the minimum clinically important difference in FJS in the 2 treatment groups was 16.5, with a power of 80% (1-β) and alpha value of 0.05 [32]. To account for a 10% loss to follow-up rate, the required sample size was 20 study patients.

### Baseline characteristics and demographics

**Fig. 1:**
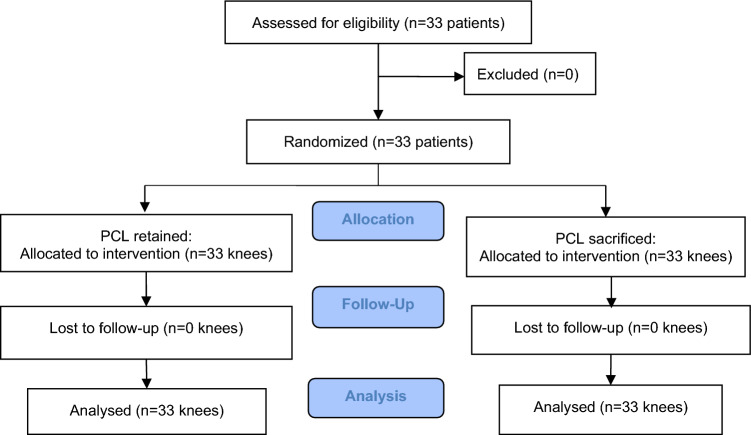
CONSORT flowchart

A total of 33 patients (66 knees) were included in this study based on the inclusion and exclusion criteria. No patient was lost to follow-up (Fig. 1). In total, 24 women and 9 men with a mean age of 71 years (range 55–90 years) and a mean body mass index (BMI) of 25.8 kg/m^2^ (range 18.3–35.6 kg/m^2^) participated in this study. Baseline characteristics were comparable in both groups (Table 1). There was no significant difference between the PCL-retained MP-TKAs (Group 1) and PCL-resected MP-TKAs (Group 2) groups in terms of preoperative VAS score, ROM, mFTA measurement, OKS, KOOS-S, or KOOS-QoL (*p* = n.s.).

**Table 1:** Baseline characteristics

	PCL retained (Mean ± SD)	PCL resected (Mean ± SD)	*p* value
VAS score	7.91 ± 0.80	8.09 ± 0.98	0.67
ROM	111.97 ± 11.59	111.21 ± 10.53	0.48
mFTA measurement	188.36 ± 2.93	188.51 ± 3.53	0.34
OKS	23.88 ± 3.29	23.30 ± 3.3	0.75
KOOS-S	40.76 ± 10.57	41.03 ± 10.49	0.96
KOOS-QoL	25.24 ± 7.96	25.48 ± 9.79	0.10

Values are expressed as the mean ± SD, with range in parentheses. Parametric data were analysed via unpaired *t* tests; nonparametric data were analysed via Mann‒Whitney *U* tests

*ROM* range of motion, *OKS*, oxford knee score, *KOOS-s* knee injury and osteoarthritis outcome score for symptoms, *KOOS-QoL* knee injury and osteoarthritis outcome score for quality of life, *SD* standard deviation

### Ethical aspects

This study was conducted according to the guidelines of the Declaration of Helsinki and approved by the Institutional Review Board of Medistra Hospital, number 004/EA/KEPKM/2017. All patients have already approved and signed the informed consent prior to enrolment in this study.

### Statistical analysis

Analysis was carried out using intention-to-treat principles. Independent samples *t* tests were used to compare demographic characteristics and study outcomes found to be normally distributed, while the Mann‒Whitney *U* test was used for continuous variables found not to be normally distributed. Categorical variables were compared using Fisher’s exact test. Variables were summarized with means and standard deviations for continuous variables and frequency counts and percentages for categorical variables with 95% confidence limits for the differences between groups at each time point. Statistical significance was set at a *P* value < 0.05 for all analyses. Analysis was performed using SPSS (Version 28.0; IBM).

## Results

### Range of motion

There was no difference in ROM at the 2-year follow-up between the PCL-retaining group and the PCL resection group (*p* = n.s.) (Table 2).

**Table 2:** Results of analysis of primary and secondary outcomes at the 2-year follow-up

	PCL retained (Mean ± SD)	PCL resected (Mean ± SD)	*p* value
VAS score	1.94 ± 0.79	2.00 ± 0.71	0.51
ROM	125.45 ± 7.00	126.21 ± 6.73	0.65
mFTA measurement	180.27 ± 2.25	181.30 ± 2.13	0.59
OKS	39.97 ± 2.01	39.67 ± 2.03	0.52
KOOS-S	84.41 ± 3.77	84.19 ± 3.57	0.92
KOOS-QoL	82.94 ± 4.76	82.75 ± 4.70	0.84

Values are expressed as the mean ± SD, with range in parentheses. Parametric data were analysed via unpaired t tests; nonparametric data were analysed via Mann‒Whitney *U* tests

*ROM* range of motion, *OKS* Oxford Knee Score, *KOOS-S* Knee Injury and Osteoarthritis Outcome Score for symptoms, *KOOS-QoL* knee injury and osteoarthritis outcome score for quality of life, *FJS* forgotten joint score, *SD* standard deviation

### Patient-reported outcome measures

Functional outcomes at the 2-year follow-up were comparable between patients with PCL retention and PCL resection (Table 2). There was no difference in VAS scores between the groups. The OKS, KOOS-S, KOOS-QoL, and FJS were the same (*p* = n.s.). The same results were also found for each FJS question (Table 3).

**Table 3:** The FJS at the 2-year follow-up

Q: Are you aware of your artificial joint when?	PCL retained (Mean ± SD)	PCL resected (Mean ± SD)	*p* value
In bed at night	0	0	0
Sitting on a chair	6.06 ± 10.88	5.30 ± 10.38	0.56
Walking for 15 min	29.55 ± 11.62	31.06 ± 12.55	0.36
Taking a bath	3.06 ± 8.28	3.79 ± 9.10	0.48
Travelling in a car	3.79 ± 9.10	3.79 ± 9.10	1.00
Climbing stairs	59.09 ± 12.21	56.82 ± 12.92	0.66
Walking unevenly	44.69 ± 14.99	43.18 ± 16.86	0.27
Standing up	44.69 ± 16.25	43.18 ± 16.86	0.59
Standing for a long period	34.09 ± 13.72	34.09 ± 13.72	1.00
Doing housework	28.79 ± 22.64	28.79 ± 22.64	1.00
Walking or hiking	40.91 ± 18.56	40.91 ± 18.56	1.00
Playing your favourite sport	37.12 ± 18.86	37.12 ± 18.86	1.00
Total score	72.66 ± 8.99	72.35 ± 8.64	0.76

Values are expressed as the mean ± SD, with range in parentheses. Parametric data were analysed via unpaired *t* tests; nonparametric data were analysed via Mann‒Whitney *U* tests

Score for quality of life, *FJS*, Forgotten Joint Score, *SD* standard deviation

### Radiographic outcomes

There was no difference in postoperative limb alignment between the PCL retention and PCL resection groups (*p* = n.s.) (Table 2).

### Complications

No complications associated with infections or joint instabilities were found in either group. No revision surgery due to pain or stiffness problems was necessary in this study.

## Discussion

The most important finding in this study was that there was no difference in ROM or functional or radiographic outcomes between the 2 treatment groups at the short-term follow-up visit. Both options are safe and give satisfactory results. This is the first study to compare PCL retention and PCL resection in simultaneous bilateral TKA with MP implants. Therefore, we eliminated confounders such as differences in pain and subjective perceptions between individuals.

There is ongoing debate on whether to retain or excise the PCL. Excision of the PCL leads to unpredictable laxity in flexion, resulting in less internal rotation of the tibia during flexion after TKA. However, PCL retention will maintain more of the tibia's internal rotation with the knee in flexion, optimizing patellofemoral tracking, but with a tighter flexion gap as a trade-off. Restoring native internal rotation of the tibia could minimize the risks of patellar tilt, lateral displacement, and anterior knee pain, which is needed to achieve high satisfaction after TKA. However, maintaining adequate flexion space could prevent loss of passive internal rotation of the tibia relative to the femur and anterior lift-off of the insert [23]. Several kinematic studies have demonstrated the effects of preserving and resecting the PCL in MP-TKA. A study of ten cadaveric knees showed that PCL retention restored more passive internal tibial rotation than PCL excision with a negligible risk of anterior lift-off. Proper tensioning of the PCL was also required to promote native knee internal tibial rotation in PCL retention [24]. However, this was a study of MP-TKA with kinematic alignment in a small number of patients. Another in vivo kinematic study of 17 clinically successful MP-TKAs in patients during stair climbing showed that knees with intact PCLs showed significantly greater tibial internal rotation than PCL-resected knees in flexion at 30° and greater. Regardless of whether the PCL was preserved or resected, patients who underwent MP-TKA had medial pivot motion patterns during stair climbing activities [17].

The anteroposterior translation of the femur might influence range of motion. PCL preservation will result in better femoral rollback, which will increase the range of motion of CR implants [13]. However, it is unclear whether retaining the PCL becomes an obstacle to lateral femoral rollback during medial pivot motions. In this study, we did not find any difference in the range of motion of knees with a preserved or resected PCL. This may be more affected by the surgical technique [22], proper tensioning of the collateral ligament and adequate flexion gap tightness achieved. When we found flexion tightness, the posterior tibial slope was increased in knees without a resected PCL. The goal is to achieve a balance between flexion and extension gaps. Another reason may also be due to the design of more constraints on the medial side with high congruency for the medial compartment to provide anterior–posterior stability combined with unrestricted motion of the lateral compartment. The lateral side moves front and back, rotating with the medial side as the centre during flexion and extension. The raised anterior lip in the MP design also stabilizes the knee from full extension through maximum flexion, confining the anterior sliding with a greater anterior constraint and subluxation resistance. With this system, it does not require an intact PCL [9].

Good patient-reported outcome measures (PROMs) were reported with medial pivot prostheses [2, 6, 9, 22, 29]. Our study’s results are similar to those of a previous study that showed no difference in PROMs between PCL retention or resection in MP-TKA [11]. Therefore, retaining or resecting the PCL did not affect the PROMs as long as appropriate PCL tension, collateral ligament tension, and balance of the flexion gap could be achieved.

The FJS was considered the best measurement to evaluate high-end functionality post arthroplasty, and “forgetting the joint” may be the ultimate goal of arthroplasty [16, 20, 28, 33, 34]. In our study, we found no difference between the MP-PCL retain group and the MP-PCL sacrifice group. These results were similar to those of a previous study [35]. This may be due to the normal kinematics that were mimicked by the MP implant. A previous study showed that either the PCL or the post-cam mechanism is necessary for medial pivot implants to regain normal kinematics [10]. Additionally, we performed simultaneous TKA, which eliminated confounders such as differences in pain and subjective perceptions between individuals. Therefore, the subjective FJS can be excluded.

Based on this study’s results, retaining PCL in medial pivot TKA is safe and comparable with sacrificing the PCL. Nevertheless, this study has several limitations. First, the patient sample size was small and the follow-up period was short. However, this study was a double-blind study of bilateral simultaneous TKA. Comparing clinical outcomes to the contralateral knees in the same individual in simultaneous bilateral TKA could eliminate confounders such as differences in pain and subjective perceptions between individuals. Secondly, our study did not undergo age and gender matching, while in the previous studies, gender is associated with residual pain, which is still controversial, and age at surgery is also associated with residual pain. Thirdly, the same prosthetic implant was used in all patients. Different MP designs from other manufacturers might give different results. Finally, this study only included knees with varus deformities and excluded those with valgus deformities. Valgus deformity might have different effects due to different kinematics.

## Conclusions

Medial pivot TKA with PCL retention and with PCL resection yielded excellent results in this study. The medial pivot implant can be used with or without the PCL with excellent results but should be placed using a surgical technique that maintains a balanced flexion gap.

## Data Availability

The data that support the findings of this study are available from the corresponding author upon reasonable request.

## Abbreviations

BMI: Body mass index
CR: Cruciate-retaining
FJS: Forgotten Joint Score
KOOS-S: Knee Injury and Osteoarthritis Outcome Score for symptoms
KOOS-QoL: Knee Injury and Osteoarthritis Outcome Score for quality of life
mFTA: Mechanical femoral-tibial axis
MP: Medial pivot
MP-TKA: Medial pivot total knee arthroplasty
OA: Osteoarthritis
OKS: Oxford knee score
PCL: Posterior cruciate ligament
PROMs: Patient-reported outcome measures
PS: Posterior-stabilized
ROM: Range of motion
TKA: Total knee arthroplasty
VAS: Visual analogue scale
